# Mussel-Inspired Hydrogels Incorporating Graphite Derivatives for Soft Tissue Regeneration

**DOI:** 10.3390/nano15040276

**Published:** 2025-02-12

**Authors:** Filipa Fernandes, Daniela Peixoto, Cátia Correia, Magda Silva, Maria C. Paiva, Natália M. Alves

**Affiliations:** 1 3B’s Research Group, I3Bs—Research Institute on Biomaterials, Biodegradables and Biomimetics, University of Minho Headquarters of the European Institute of Excellence on Tissue Engineering and Regenerative Medicine, Avepark, 4805-694 Guimarães, Portugal; pg38293@alunos.uminho.pt (F.F.); daniela.peixoto@i3bs.uminho.pt (D.P.); catia.correia@i3bs.uminho.pt (C.C.); 2 ICVS/3B’s, Associate PT Government Laboratory, 4710-057 Braga, Portugal; 3 Department of Polymer Engineering, Institute for Polymers and Composites, University of Minho, 4800-058 Guimarães, Portugal; id6877@uminho.pt

**Keywords:** hyaluronic acid, graphite, biomimetic approach

## Abstract

Hyaluronic acid (HA)-based hydrogels offer a promising approach for soft tissue application due to their biocompatibility, tunable mechanical properties, ability to mimic the extracellular matrix, and capacity to support cell adhesion and proliferation. In this work, bioadhesive composite hydrogels were developed by integrating graphite derivatives (EG) into a dopamine-modified HA matrix (HA-Cat), which enhances tissue adhesion through catechol groups that mimic mussel-inspired adhesion mechanisms. The EG was functionalized via 1,3-dipolar cycloaddition reaction (f-EG), that allowed the anchoring of silver nanoparticles (f-EG-Ag) and grafting of hydrocaffeic acid (f-EG-Cat) on the functionalized EG surfaces. The hydrogels were produced by oxidative crosslinking of HA-Cat under mild basic pH conditions using sodium periodate. Indirect in vitro assays using L929 fibroblast cells showed high biocompatibility and enhanced cell proliferation at optimized composite hydrogel concentrations. These findings suggest that composite hydrogels could find an application as bioactive, adhesive scaffolds for the regeneration of soft tissues, where they can facilitate localized agent delivery and integration with the host tissue.

## 1. Introduction

Hydrogels are versatile, highly hydrated three-dimensional polymeric networks extensively employed in biomedical applications due to their ability to mimic the extracellular matrix, biocompatibility, and tunable physical properties [1,2]. Among these, hyaluronic acid (HA)-based hydrogels have advanced particular attention as scaffolds for soft tissue engineering and regenerative medicine.

Hyaluronic acid (HA) is a naturally occurring, long-chain polysaccharide widely recognized for its structural and functional versatility in biological systems. Composed of alternating glucuronic acid and *N*-acetyl-glucosamine units linked by β-(1,3) and β-(1,4) glycosidic bonds, HA adopts a helical conformation due to its chemical structure and intramolecular hydrogen bonding. This unique configuration results in hydrophilic and hydrophobic segments along its backbone, which contribute to its diverse interactions with biological molecules [2,3,4]

When hydrogels are implanted in the human body, the interfacial adhesion between the polymeric matrix and the substrate is hindered by the moist environment, leading to the misplacement of the hydrogel [5,6,7,8]. To tackle this problem, material scientists have taken inspiration from natural phenomena. Particularly, marine mussels have developed an effective strategy to firmly adhere to different surfaces under wet conditions [9], resisting the movements of tides and currents. The adhesive proteins secreted by them are characterized by high amounts of the amino acid 3,4-dihydroxyphenylalanine (DOPA) [9,10] and, specifically, the catechol moieties are responsible for mediating both interfacial adhesion to organic and inorganic surfaces and the cohesion of the adhesive proteins [11,12,13].

The incorporation of functionalized nanomaterials into hydrogels is a promising strategy to improve their mechanical, biological, and functional properties [14,15]. Graphite derivatives, due to their unique surface chemistry and structural characteristics, have been widely explored as fillers to reinforce polymer matrices.

The present work investigated the influence of the incorporation of a nano-graphite derivative (EG) in mussel-inspired self-adhesive hyaluronic acid hydrogels. The EG was functionalized via the 1,3-dipolar cycloaddition of an azomethine ylide, forming pyrrolidine groups that allowed the anchoring of silver nanoparticles and grafting of hydrocaffeic acid. The final hydrogels were produced by oxidative cross-linking of catechol-modified hyaluronic acid under mild basic pH conditions and in the presence of sodium periodate. The resulting hydrogel composites exhibit favorable mechanical behavior, cytocompatibility, and antibacterial activity, positioning them as promising candidates for soft tissue regeneration.

## 2. Materials and Methods

### 2.1. Materials

Sodium hyaluronate (molecular weight-MW = 351–600 kDa) was provided by LIFECORE BIOMEDICAL, Chaska, MN, USA. Dopamine hydrochloride, *N*-(3-(dimethylamino)propyl)-*N*′-ethylcarbodiimide hydrochloride (EDC), *N*-hydroxysuccinimide (NHS), sodium periodate (NaIO_4_), deuterium oxide (D_2_O), phosphate buffered saline (PBS), *N*-benzyloxycarbonylglycine (Z-Gly-OH), paraformaldehyde (PFA), and silver nitrate (AgNO_3_) were purchased from Sigma-Aldrich, Buchs, Switzerland. Dialysis membrane Spectra/Por 3 (cut-off 3.5 KDa, width 54 mm, diameter 34 mm) was obtained from Fisher Scientific, San Francisco, CA, USA. Sodium Hydroxide pellets (NaOH), sodium chloride (NaCl), and ethanol Absolut (EtOH) were obtained from Honeywell Specialty Chemicals Seelze, Germany. Micrograf HC11 (hereafter referred to as EG), a graphite material subjected to grinding and exfoliation, was purchased from Nacional de Grafite Lda, Itapecerica, MG, Brazil, with a purity degree of 99.5%, an average particle size (d50) of 11.0 μm, and an estimated thickness in the range of 15–30 nm. Mouse fibroblast cell line L929 was obtained from Sigma-Aldrich partnered with the European Collection of authenticated cell cultures (ECACC, Salisbury, UK). Dubecco’s modified minimum essential medium (DMEM) low glucose with and without phenol red, and sodium bicarbonate (NaHCO_3_) were purchased from Sigma-Aldrich (St. Louis, MO, USA). Antibiotic/antimycotic, Dulbecco’s Phosphate Buffered Saline (DPBS), trypLE express (1x) with phenol red, fetal bovine serum (FBS), Calcein AM and propidium iodide (PI) were ordered from Thermo-Fisher Scientific (Bleiswijk, The Netherlands). CellTiter 96^®^ AQueous One Solution Reagent was purchased from Promega (Fitchburg, WI, USA).

### 2.2. Functionalization of the EG

In order to enhance the dispersion and the filler–matrix interface, taking into account the hydrophobic nature of the EG, a functionalization procedure was carried out based on the one pot, solvent-free approach to the 1,3-dipolar cycloaddition of an azomethine ylide. The procedure was adapted from a previous work described in the literature for carbon nanotubes [16]. First, 10 g of Z-Gly-OH were suspended in 50 mL of diethyl ether, under magnetic stirring for 10 min at room temperature. Then, 7 g of PFA was added to the suspension, under the same stirring conditions. After that, 10 g of EG and 50 mL of diethyl ether were added to the previous suspension. The suspension was gently heated until the solvent was completely removed and Z-Gly-OH and PFA formed a homogeneous mixture with the EG. The solid mixture was then heated at 250 °C for 3 h, in a two-neck round-bottom flask. The product was re-dispersed in ethanol by sonication for 15 min, and the suspension was filtered and washed with a sequence of solvents, namely hexane, ethanol, and acetone. The resulting functionalized EG (f-EG) was dried under vacuum, at 150 °C for 3 h. F-EG was obtained as a black solid (Figure S1a).

### 2.3. Anchoring Silver Nanoparticles on Functionalized EG

Considering the well-known antibacterial properties of silver, the anchoring of silver nanoparticles at the f-EG surface was carried out to endow the final hydrogel with antibacterial properties. This anchoring was based on the reduction of AgNO_3_ in DMF [17]. Primarily, 280 mg of f-EG were dispersed in 16 mL of DMF and magnetically stirred for 15 min. Meanwhile, suspensions of 140 mg of AgNO_3_ in 8 mL of EtOH were kept under magnetic stirring for 15 min and mixed with the previous suspensions of f-EG. The mixtures were then stirred for 72 h with an ultrasonication step (15 min, ultrasonic bath) every 24 h. The reaction flasks were covered with aluminum foil throughout the procedure to avoid oxidation. Finally, the reaction products were filtered and washed with hexane followed by diethyl ether. The resulting f-EG decorated with silver nanoparticles (f-EG-AgNP) were dried under vacuum, at 150 °C for 2 h. F-EG-AgNPs was obtained as a black solid (Figure S1b).

### 2.4. Synthesis of Catechol Conjugated with Functionalized EG

Taking advantage of the ability of catechol to establish multiple chemical or physical interactions, the possible bonding of this group to the f-EG surface was investigated, aiming at an enhanced interaction between the filler and the catechol groups of dopamine-modified HA that constitute the hydrogel matrix. Chemically modified f-EG with catechol groups (f-EG-Cat) was prepared by conjugating hydrocaffeic acid to the pyrrolidine group of f-EG, through a carbodiimide coupling reaction using EDC and NHS as activation agents of the carboxyl groups on hydrocaffeic acid. Briefly, 250 mg of f-EG was dissolved in 25 mL of PBS solution, and the pH was adjusted to 5.5 using HCl (1 M). Then, the solution was purged with nitrogen for 30 min, to perform the reaction in an inert atmosphere. After that, 436.34 mg of hydrocaffeic acid, 371.83 mg of EDC and 275.65 mg of NHS were added, and the pH of the reaction solution was maintained at 5.5 for 2 h. Reaction products were centrifuged three times at 9000 rpm for 10 min, intercalating washing steps with deionized water. The resulting f-EG conjugated with catechol groups (f-EG-Cat) was dried overnight at 40 °C, under vacuum. F-EG-Cat was obtained as a black solid (Figure S1c). The f-EG-Cat was analyzed by ^1^H nuclear magnetic resonance in deuterium water (NMR, 400 MHz, D_2_O).

### 2.5. Synthesis of Dopamine-Conjugated Hyaluronic Acid

Chemically modified hyaluronic acid with catechol groups (HA−Cat) was prepared by conjugating dopamine hydrochloride to HA, through a carbodiimide coupling reaction using EDC as an activation agent of the carboxyl groups on HA chains. The procedure was based on previous works described in the literature [18,19]. Primarily, 1 g of HA was dissolved in 100 mL of PBS solution, followed by adjusting the pH to 5.5 using HCl aqueous solution. Then, the solution was purged with nitrogen for 30 min, to perform the reaction in an inert atmosphere. Subsequently, 504 mg of EDC and 711 mg of dopamine hydrochloride were added, and the pH of the reaction solution was maintained at 5.5 for 2 h. Unreacted chemicals and urea byproducts were removed by extensive dialysis for a week. Afterwards, the conjugate was frozen at −80 °C and lyophilized. To avoid oxidation, the conjugate was stored at 4 °C and protected from the light. The resultant HA−Cat conjugate was analyzed by ^1^H NMR (400 MHz, D_2_O, Bruker Avance III, Wissembourg, France) and UV-vis spectroscopy (BIO-TEK instruments, Winooski, VT, USA) was used to calculate the degree of substitution in the HA backbone by quantifying the absorbance at 280 nm which is characteristic of catechol. The molecular weight was calculated by gel permeation chromatography (GPC) (Malvern Viscotek TDA 305) with a refractometer (RI-Detector 8110, Bischoff, Paralab, Giesta, Valbom).

### 2.6. Characterization of the EG Derivatives

Raman spectroscopy analysis of the EG derivatives was performed on a LabRAM HR Evolution Raman spectrometer with a microscope (Horiba Scientific, Piscataway, NJ, USA). The measurements were made with a laser excitation wavelength of 532 nm. The results were analyzed using Horiba Scientific´s LabSpec 6 (version 6.4.4) software.

The surface chemistry of the EG derivatives was characterized using an X-ray photoelectron spectroscope (Axis supra, Kratos, Manchester, M17 1GP, UK) equipped with monochromatic AI-Kα radiation at 1486.6 eV, within ESCape software. Each measurement was conducted in a Constant Analyzer Energy mode (CAE) with a 160 eV pass energy for survey spectra and 40 eV pass energy for high-resolution spectra. Data analysis and atomic concentration were determined from the X-ray photoelectron spectroscopy (XPS) peaks of high-resolution scans using ESCape software (version 1.4) supplied by Kratos analytical.

### 2.7. Preparation and Characterization of HA-Cat Composite Hydrogel

The hydrogels were produced by oxidative cross-linking of the catechol moieties of HA-Cat under mild basic pH conditions and in the presence of NaIO_4_ [12,20]. Different concentrations of HA-Cat conjugate and oxidant agents were tested (Table S1 and Figure S2). Afterwards, the conditions used in the preparation of sample 27 were selected, i.e., 10 mg of HA-Cat was dissolved in 100 µL PBS at pH 8–9 and then 0.25 mg of NaIO_4_ was added, to combine the polymer and the reinforcement.

The cross-section morphology of freeze-dried HA-Cat hydrogels was investigated by scanning electron microscopy (SEM, JSM-6010LV scanning electron microscope (JEOL, Japan, Akishima City, Tokyo, Japan)). Before observation, the surface of the lyophilized hydrogels was coated with a gold layer, except for the hydrogels containing f-EG-AgNP.

Rheological analyses were performed utilizing a Kinexus pro+ rheometer (Malvern Instruments, Malvern WR14 1XZ, UK), and the respective Malvern’s acquisition software: rSpace. The measuring plate-plate system used was equipped with an upper measurement geometry (8 mm of diameter) and a lower plate pedestal, both with stainless steel (316 grade) and a rough finish. The linear viscoelastic region (LVER) for the hydrogels was determined through a strain sweep test (0.1–100%) at a constant frequency (1.0 Hz) and temperature (25 °C). The samples’ mechanical spectra (frequency sweep curves) were obtained at a fixed temperature of 25 °C from 0.1 Hz to 10 Hz of frequencies to study the structures’ viscoelastic properties. All plots were built using an average of three experiments.

### 2.8. Biological Assays

Indirect cytotoxicity of the composite hydrogels was analyzed by MTS assay and calcein/PI staining using L929 fibroblastic cells, at 1, 3, and 7 days. Firstly, the hydrogels were sterilized by UV for 1 h. Then, 70 mg of each hydrogel was placed in a 15 mL sterile tube with 2 mL of complete cell culture medium in triplicate. The tubes were placed in a water bath at 37 °C and 60 rpm for 24 h.

L929 mouse fibroblast cells (NCTC clone 929, ATCC^®^ CCL-1™, passage 42) were cultured in DMEM low glucose supplemented with 10% FBS and 1% of an Antibiotic-Antimycotic solution. Cells were grown in a T150 flask and incubated at 37 °C in a humidified air atmosphere of 5% CO_2_. The medium was changed every 2 days. Upon 80% of confluence, the cells were washed with DPBS and subsequently detached with 5 mL of trypLE™ express solution for 5 min at 37 °C. To inactivate the trypLE™, 10 mL of culture medium was added. The cells were centrifuged at 300 rcf for 5 min and the obtained pellet was resuspended in the culture medium.

The cells were seeded in a 96-well plate using a cell suspension of 10,000 cells per well. The well-plate was incubated for 24 h in a humidified air atmosphere of 5% CO_2_ to establish a 70–80% monolayer. After 24 h of culture, the culture medium was removed and was replaced by 100 μL of complete cell culture medium that was previously in contact with hydrogels in different concentrations (35, 17.5, and 8.75 mg/mL) after being passed through a 0.45 μm membrane filter.

The MTS assay was used to indirectly analyze the cytotoxicity of different concentrations of the composite hydrogels. At each time-point (24, 48, and 72 h), the culture medium was removed, and the wells were washed with 100 µL of PBS. Then, a mixture of MTS medium and MTS reagent containing serum-free cell culture medium without phenol red and CellTiter 96^®^ AQueous One Solution Reagent (5:1 ratio), was added to each well. The 96-well plate was covered with aluminum foil and incubated for 3 h in a humidified air atmosphere of 5% CO_2_. At the end of the incubation period, 100 μL of each well was transferred to a new 96-well plate and the absorbance was measured at 490 nm using a microplate reader (BIO-TEK Instruments, Winooski, VT, USA).

The live/dead assay was used to indirectly analyze the viability of L929 fibroblastic cells after being in contact with different percentages of cell culture medium that was previously in contact with composite hydrogels. The live/dead assay was performed using Calcein AM and PI staining. After 24, 48, and 72 h of culture, the culture medium was removed from each well and washed with 100 μL PBS. Then, 100 μL of DMEM medium was supplemented with 1 μg of Calcein AM and 0.5 μg of PI was added to each well. These immersions lasted for 15 min in the dark and were then washed with PBS and analyzed using a Fluorescence Inverted Microscope (Axio Observer, Zeiss, Jena, Göttingen, Germany).

## 3. Results

### 3.1. Production and Characterization of EG Derivatives

**Figure 1:**
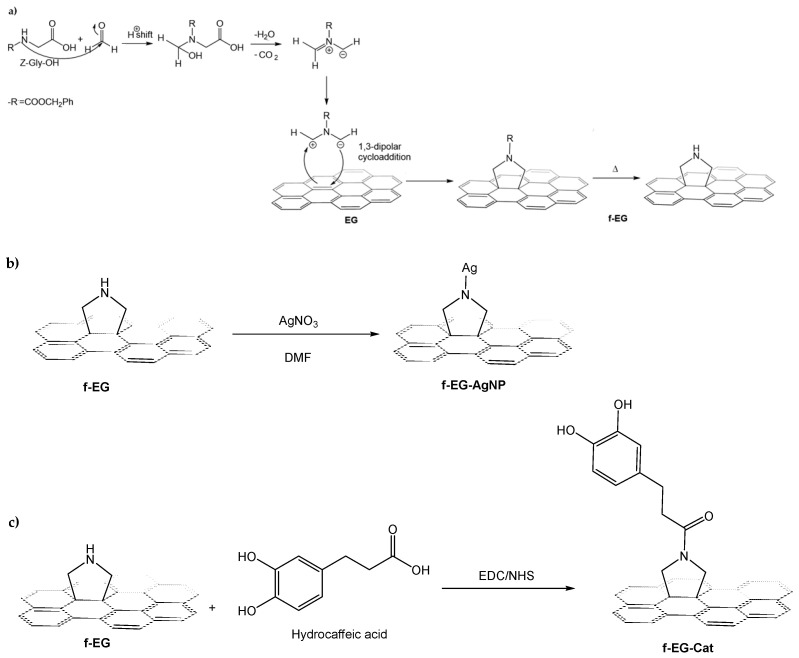
(**a**) Mechanism of 1,3-dipolar cycloaddition reaction of Z-Gly-OH and PFA on the surface of EG. (**b**) Deposition of silver nanoparticles onto f-EG surface based on the reduction of AgNO_3_ in DMF. (**c**) Scheme of conjugation of hydrocaffeic acid to the pyrrolidine group of f-EG.

The EG derivatives, namely f-EG, f-EG-AgNP, and f-EG-Cat, were successfully produced (Figure 1a–c). The functionalization approach aimed to bond pyrrolidine groups (cyclic amine) to the EG surface via cycloaddition of an azomethine ylide. The azomethine ylide was obtained by the reaction of Z-Gly-OH and formaldehyde (formed by thermal decomposition of PFA) yielding the 1,3-dipole represented in Figure 1a, that readily reacts with the EG surface. The cyclic amine formed on the EG surface allows the anchoring of Ag nanoparticles, as well as the reaction with carboxyl groups, and strengthens the interfacial bonding between the filler and the polymer matrix. The aim of forming f-EG-AgNP is to confer additional antibacterial activity at a low and controlled Ag concentration. In particular, to our knowledge, the incorporation of catechol groups in f-EG was conducted for the first time, as it was hypothesized that it could also promote an enhanced interaction between the filler and the catechol groups of dopamine-modified HA that constitute the hydrogel matrix. After the functionalization procedures were carried out, the structural characterization of each EG derivative was conducted by Raman Spectroscopy and XPS.

#### 3.1.1. Raman Spectroscopy

**Figure 2:**
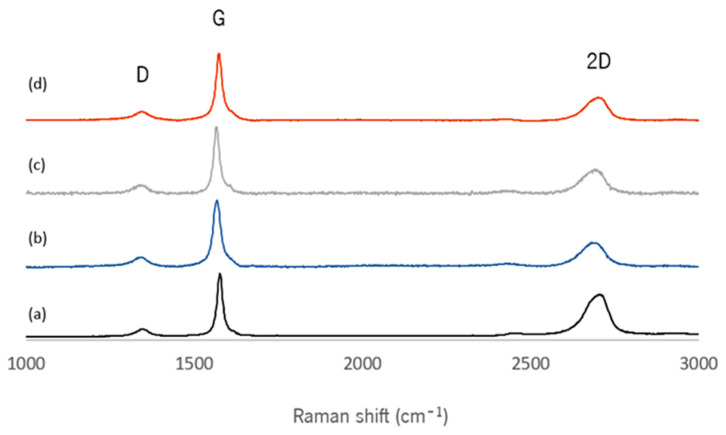
Raman spectra of (**a**) EG, (**b**) f-EG, (**c**) f-EG-AgNP and (**d**) f-EG-Cat samples.

The spectra presented in Figure 2 of each EG derivative exhibit two main peaks at ~1565–1576 and ~2682–2705 cm^−1^, namely G and 2D (or G′) bands, respectively. A minor D band, whose intensity is proportional to the extent of disorder in the graphite structure [21], is also detected at ~1342–1347 cm^−1^. These constitute the typical Raman bands for graphitic materials. The spectrum of a single layer of graphene typically depicts a symmetrical 2D band with higher intensity compared to the G-band [22,23]. As the number of layers increases, the intensity of the 2D/G ratio decreases [24], and the 2D band splits and shifts to higher wavenumbers [25]. Therefore, the position, shape, and relative intensity of the Raman peaks are informative about the graphene/graphite material structure. The slight displacement of the 2D peak position to lower Raman shift values observed for the functionalized EG derivatives, relative to the pristine flakes, may be indicative of a higher degree of exfoliation of the functionalized nanomaterials, resulting in thinner functionalized flakes [26]. Furthermore, the ID/IG ratio obtained for the pristine and functionalized materials is small, indicating a good structural quality of the materials. The ID/IG ratio remained constant for the functionalized materials compared to pristine EG, demonstrating that the structural quality of graphite was not affected by the functionalization processes, as observed by SEM analysis (Figure S3).

#### 3.1.2. X-Ray Photoelectron Spectroscopy

**Figure 3:**
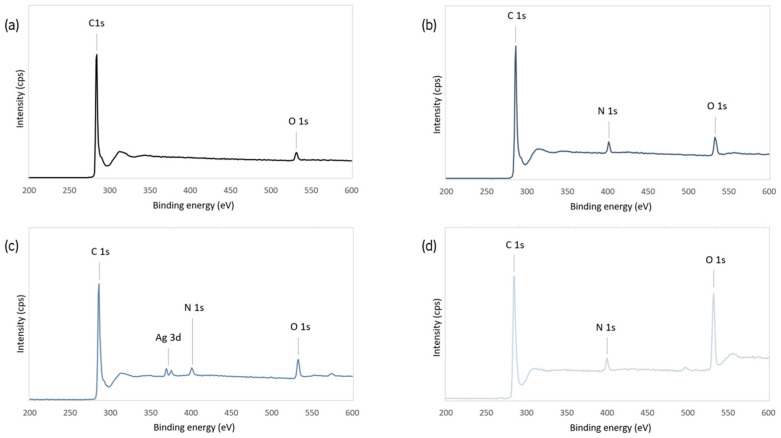
Wide-scan XPS spectra of (**a**) EG, (**b**) f-EG, (**c**) f-EG-AgNP, and (**d**) f-EG-Cat.

The surface composition of EG and its derivatives was analyzed by XPS; the respective wide-scan spectra are presented in Figure 3 and the elemental composition of EG derivatives is presented in Table 1. All EG derivatives show two peaks corresponding to the binding energy of C 1s and O 1s. After the 1,3-dipolar cycloaddition reaction, a peak corresponding to the binding energy of N 1s is observed, as well as a small increase in oxygen at.%, due to the remanence of a fraction of R groups bonded to the cyclic amine (Figure 1a. The intensity of the O 1s peak further increased by approximately 10 at.% for f-EG -Cat, indicating the successful conjugation with hydrocaffeic acid. The presence of silver was also confirmed for f-EG-AgNP.

Table 1 shows that the f-EG formed contains approximately 2.3 at.% of oxygen in excess relative to EG, and 5.3 at.% of nitrogen. Thus, approximately 20% of the nitrogen is bonded to an R group, that contains two oxygen atoms, and 80% of the nitrogen functional groups bonded to the EG surface are in the pyrrolidine form, a cyclic amine that may react with carboxyl groups. Bonding hydrocaffeic acid to the f-EG surface was effective, as demonstrated by the increase of approximately 10.6 at.% in oxygen, relative to f-EG. Considering that each hydrocaffeic acid molecule contains three oxygen atoms, this is indicative that nearly 3.5 at.% of nitrogen is engaged in this bond (almost all the pyrrolidine functional groups formed).

The high-resolution C 1s spectrum of EG (Figure S4) revealed a prominent, narrow peak at 285.02 eV, attributed to the sp^2^ hybridized carbons of the graphite lattice, and a weak satellite (291.71 eV), assigned to the conjugated π-electrons in the aromatic system. A second peak due to sp^3^ hybridized carbons was observed at 285.34 eV, while other contributions appeared as component peaks at 286.75 and 289.29 eV, probably due to functional groups at the edges and defects of the graphite sheets [17,27,28,29,30]. After the cycloaddition reaction, the peak corresponding to the sp^3^ hybridized carbons increased for all samples, as a result of the bonding of the cyclic amine (pyrrolidine) to the surface of EG, changing the hybridization state of the carbon atoms. Furthermore, the appearance of peaks assigned to carbon–nitrogen and carbon–oxygen bonds (like Ph–OH or catechol, Ph–CH_2_–O–, C–N–C, O=C, O=C–O–, O=C–NH) was more pronounced, supporting the bonding of these functions on the surface of the EG derivatives [17,27].

The high-resolution O 1s spectrum of pristine EG (Figure S4a), containing an atomic concentration of approximately 2.5% of oxygen, corresponds to a different chemical composition relative to f-EG. Furthermore, the O1s spectra of EG derivatives (Figure S4b–d) support the coexistence of phenolic -OH, quinone, and carbonyl O=C, O=C–O– and O=C–NH groups, resulting from the functionalization and hydrocaffeic acid coupling reactions [28,29,31,32,33].

Figure S4b–d shows the high-resolution N 1s spectra of the products of the functionalization reaction. The deconvolution yielded two peaks: a main one corresponding to the substituted pyrrolidine group, with benzyl carbamate or hydrocaffeic acid, and another peak corresponding to -NH, as in pyrrolidine [16,17].

Finally, the Ag 3d high-resolution XPS spectrum of f-EG-AgNP (Figure S4c) shows well-separated (Δ~6.0 eV) and asymmetric peaks at binding energies of 368.91 eV for Ag 3d5/2 and 374.90 eV for Ag 3d3/2 [17]. These are characteristic of the Ag metallic form and, therefore, are indicative of the effective reduction of silver ions for the formation of metal nanoparticles.

#### 3.1.3. NMR Spectroscopy

**Figure 4:**
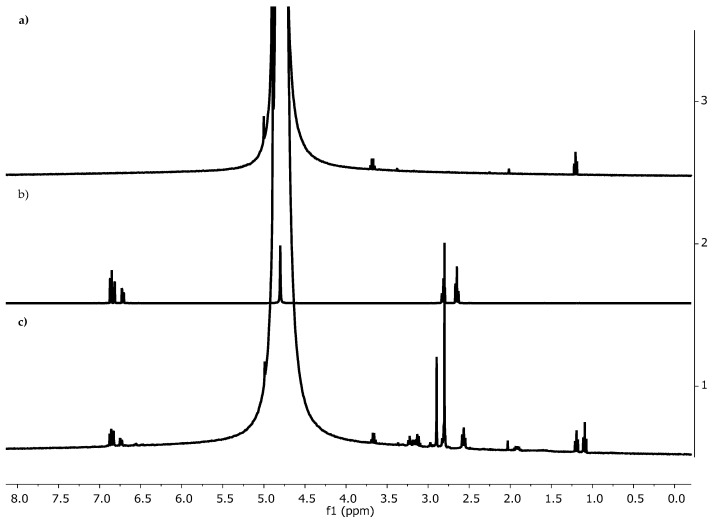
^1^H NMR (400 MHz, D_2_O) spectra of (**a**) f-EG, (**b**) hydrocaffeic acid and (**c**) f-EG-Cat.

The structure of f-EG-Cat was characterized by ^1^HNMR (Figure 4). The spectrum of f-EG-Cat exhibited signals at δ~6.5–7.0 ppm, which are absent in the spectrum of f-EG. These peaks correspond to the catechol moiety of hydrocaffeic acid, and therefore confirmed its conjugation to the pyrrolidine moiety of f-EG.

### 3.2. Production and Characterization of HA-Cat Conjugate

**Figure 5:**
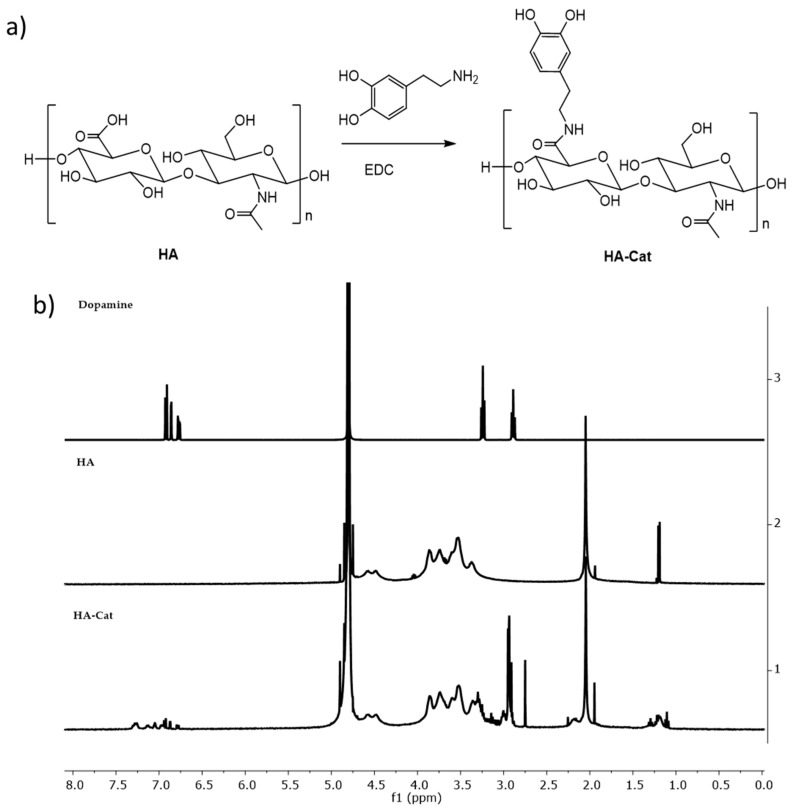
(**a**) Scheme of conjugation of dopamine to the backbone of hyaluronic acid. (**b**) ^1^H NMR (400 MHz, D_2_O) spectra of dopamine, HA, and HA-Cat.

HA was modified with catechol groups (HA-Cat) by reaction with dopamine in the presence of EDC (Figure 5a). The structure of the HA-Cat conjugate was confirmed by ^1^HNMR (Figure 5b). The spectrum of unmodified HA exhibited signals at δ~2.0 and δ~3.3–4.8 ppm that correspond to the protons of the N-acetyl group and saccharide units, respectively [19,34]. However, after the coupling of dopamine to the HA backbone, new signals at δ~2.9 and δ~6.7–7.3 ppm could be detected. These peaks emerged from the protons of the methylene group and catechol moiety of dopamine, respectively, and therefore confirmed the successful conjugation. The conjugation of dopamine onto the HA backbone was confirmed by the presence of a typical peak around 280 nm, characteristic of aromatic groups. As expected, this peak did not appear for HA, confirming the presence of dopamine only on the conjugated HA-Cat. The estimated degree of catechol substitution (DS) was around 8% (Figure S5). The MW of the HA-Cat was calculated by GPC (Figure S6) and the MW of the HA-Cat was similar to the HA (365 KDa).

### 3.3. Optimization of the Preparation of HA-Cat Hydrogels

The HA-Cat hydrogels were formed by catechol−catechol adducts under basic pH in the presence of NaIO_4_. When the pre-gel solution gelled, we checked the color change (Figure S7) [20].

Prior to the production of composite hydrogels, different formulations of HA-Cat were tested by varying the polymer concentration and amount of the oxidizing agent NaIO_4_. The vial inversion test demonstrated that most of the tried conditions did not form a stable gel, as the content in the vials flowed (Figure S2).

### 3.4. Production and Characterization of Composite Hydrogels

The EG derivatives were dispersed into the HA-Cat hydrogels. Three different methods of combining the filler and the matrix were tested (Figure S8, Table 2).

**Figure 6:**
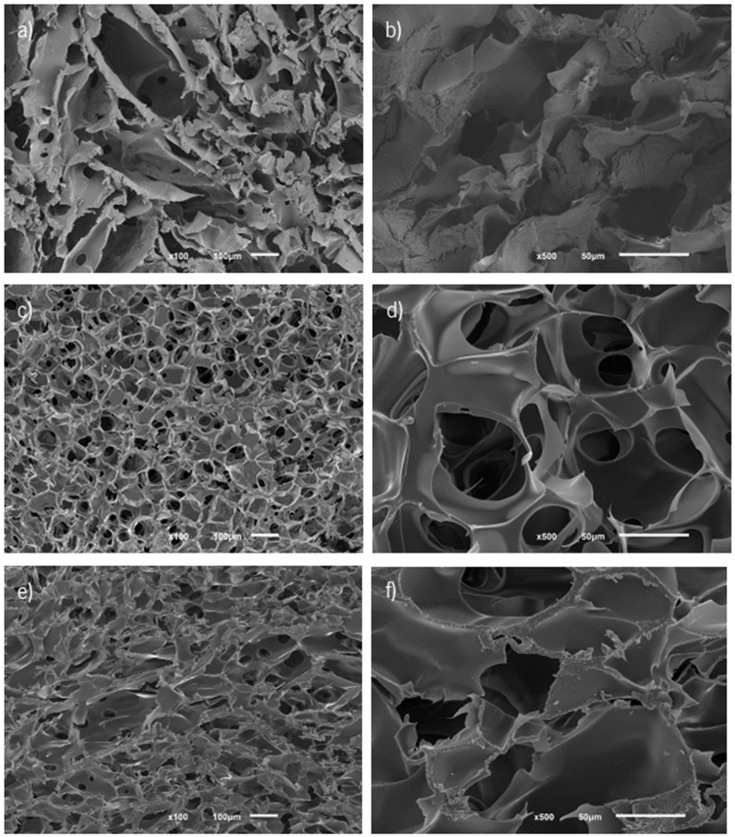
Cross-section morphology of HA-Cat reinforced with f-EG hydrogel, analyzed by SEM. (**a**,**b**) sample 1; (**c**,**d**) sample 2; (**e**,**f**) sample 3. The scale bar is 100 µM (**a**,**c**,**d**) and 50 µM (**b**,**d**,**e**).

SEM analyzed the HA-Cat combined with f-EG hydrogels (Figure 6). The method used in the preparation of sample 2 was selected and used in the subsequent characterization, since the cross-sectional SEM images of the sample revealed more interconnected and homogeneously distributed pores in its structure, compared to the other methods of mixture. Furthermore, because the pre-gel solution was highly viscous the method used in the preparation of sample 1 was challenging.

**Figure 7:**
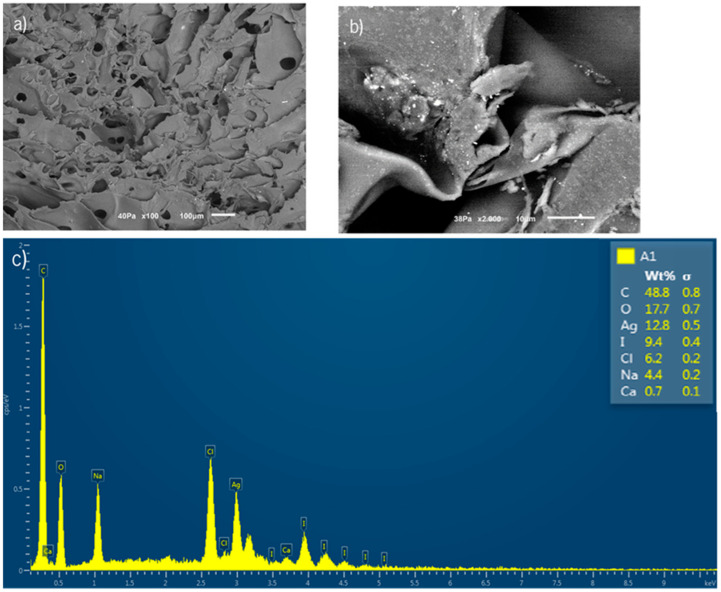
Cross-section morphology of HA-Cat reinforced with f-EG-AgNP hydrogel, analyzed by (**a**,**b**) SEM and (**c**) EDS analysis of the elements in the hydrogel. The scale bar is 100 µM.

When the HA-Cat reinforced with f-EG-AgNP hydrogel was prepared following method 2, the cross-section morphology of the resultant composite (Figure 7) clearly showed that the filler remained within the polymeric matrix, indicating a good interaction between the filler and the polymer.

**Figure 8:**
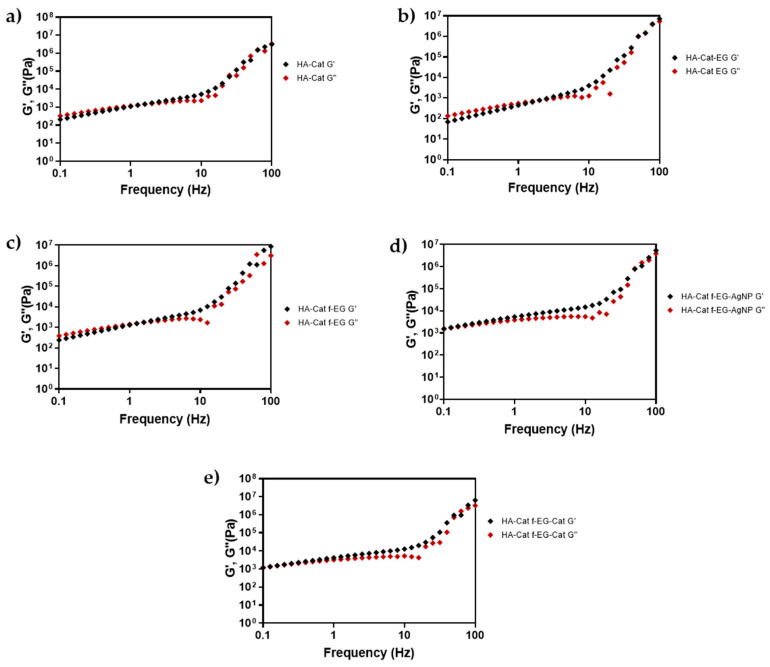
Frequency sweep strain controlled with G′ and G″ cross-over analysis for (**a**) HA-Cat hydrogels, (**b**) reinforced with EG, (**c**) f-EG, (**d**) f-EG-AgNP, and (**e**) f-EG-Cat.

The rheological behavior of freeze-dried and rehydrated composite HA-Cat hydrogels was studied through a frequency sweep test (Figure 8). All tested samples showed a frequency-dependent behavior, with the moduli increasing gradually over time, possibly due to reversible bonds between the catechol groups. As the frequency increases, and therefore the oscillation period is shortened, the polymer chains might be unable to disentangle and rearrange themselves, resulting in a “solid-like” or elastic behavior [35]. The average storage moduli measured at 10 Hz were nearly 5, 4, 7, 15, and 13 kPa for HA-Cat and HA-Cat reinforced with EG, f-EG, f-EG-AgNP, and f-EG-Cat hydrogel, respectively, indicating that the hydrogels reinforced with EG-AgNP and f-EG-Cat were more rigid than the other hydrogels [36], possibly because of a better interfacial binding between the catechol groups of HA chains and the functional groups of incorporated fillers. Furthermore, the addition of f-EG did not severely impact the rheological behavior of the hydrogel when compared with HA-Cat without reinforcement. Conversely, the addition of EG shifted the G′ and G″ crossover to a higher frequency, whereas the addition of f -EG-AgNP and f-EG-Cat shifted the G′ and G″ crossover to lower frequencies. Given the moduli values, the hydrogels could find application as a scaffold for soft-tissue engineering.

**Figure 9:**
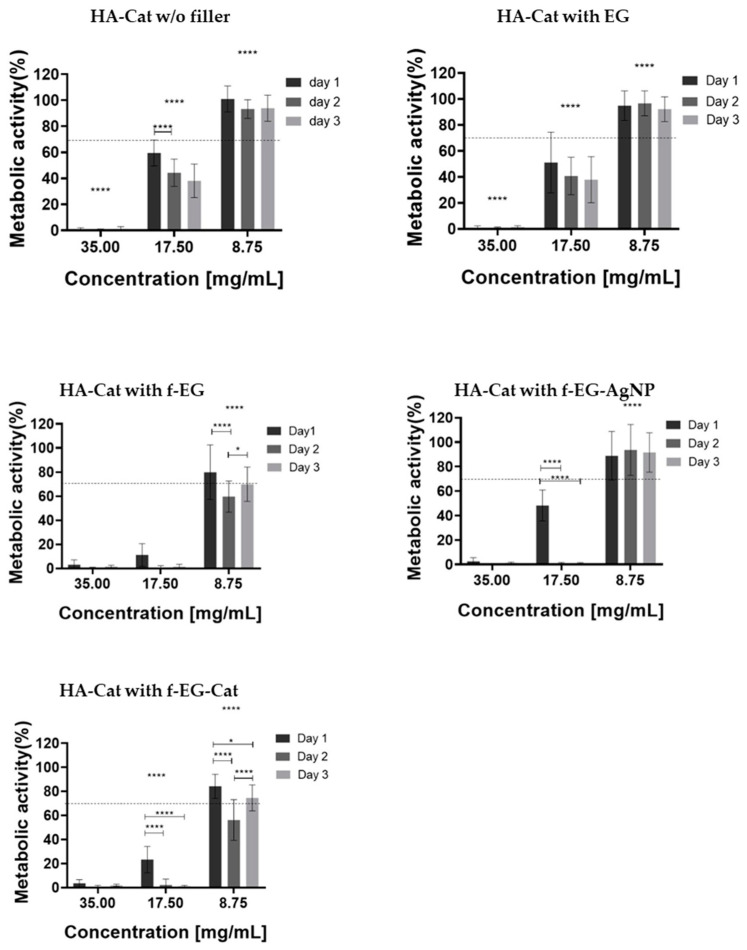
Metabolic activity of L929 fibroblast cells after treatment with cell culture mediums that were previously in contact with composite hydrogels in different concentrations (35, 17.5, and 8.75 mg/mL). Results are expressed as mean ± standard deviation where n = 12, * *p* < 0.05 and **** *p* < 0.0001, two-way ANOVA with Tukey multiple comparison post-test.

**Figure 10:**
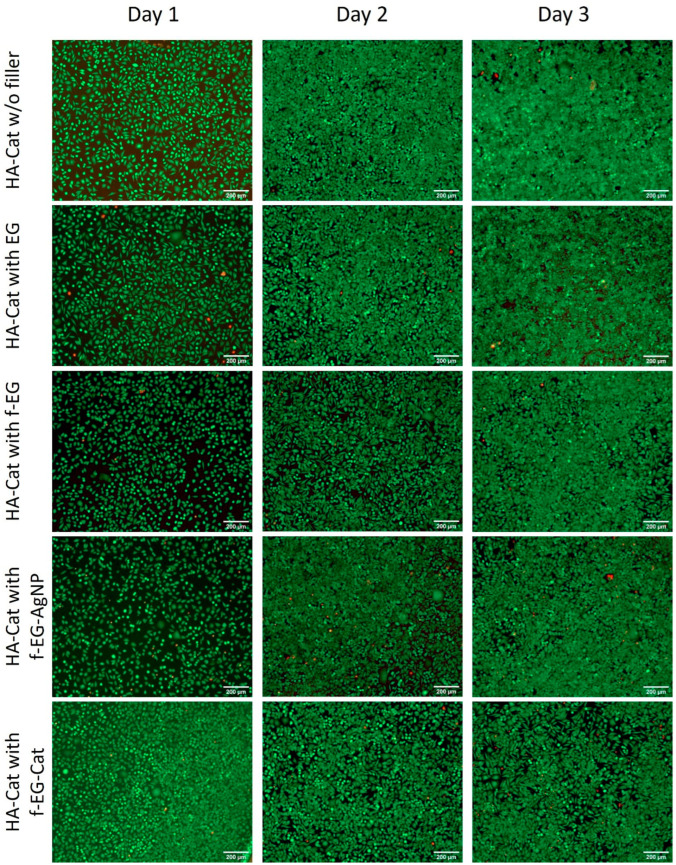
Live/dead fluorescence images of L929 fibroblast cells after incubation with a cell culture medium that was previously in contact with 8.75 mg/mL of HA-Cat hydrogels incorporated with different graphite derivatives. The scale bar is 200 µM.

The composite hydrogels were developed for application in soft tissues; therefore. we tested the cytotoxicity of hydrogels and evaluated the effect of the different fillers on cell viability. L929 mouse fibroblast cells were used. The cytotoxicity of the composite hydrogels was analyzed by MTS assay after in vitro culture of L929 fibroblast cells with different concentrations of composite formulations [37,38]. As shown in Figure 9, the highest concentration of 35 mg/mL inhibited cell growth for all the samples tested. In turn, for a concentration of 17.5 mg/mL, the addition of EG did not significantly influence the cytotoxicity, while the addition of the other EG derivatives increased it. Finally, the lowest concentration tested (8.75 mg/mL) showed better metabolic activity percentages compared with the previous values. Therefore, the addition of EG and f-EG-AgNP did not significantly influence the cytotoxicity, but the addition of f-EG and f-EG-Cat induced a slight increase in the cytotoxicity. Cell viability was further tested using the Live/Dead assay. Calcein-AM permeates the cell membrane and stains viable cells, whereas Propidium Iodide stains dead cells through the disordered cell membrane. In a fluorescence microscope, this results in the emission of green and red fluorescence, respectively, permitting the simultaneous observation of viable and dead cells. Live/dead fluorescence images of L929 fibroblast cells were consistent with the results of the MTS assay. For the higher concentration of hydrogel formulations tested (35 mg/mL), few cells were observed and most of them were stained red, indicating a high level of cytotoxicity (Figure S9). Furthermore, all samples presented detached fibroblast cells with a round morphology (Figure S10). In comparison, for a concentration of 17.5 mg/mL, only HA-Cat hydrogels reinforced with EG or in the absence of a filler showed viable cells, whereas the other formulations exhibit cytotoxicity (Figures S11 and S12). Finally, after the incubation of fibroblast cells with 8.75 mg/mL of hydrogel formulations, all samples showed an increase in cell proliferation and mainly contained viable cells stained green, indicating good biocompatibility (Figure 10). By this time, fibroblast cells had adopted a more elongated or fusiform morphology (Figure S13).

## 4. Conclusions

The successful development of multifunctional hydrogels integrating graphite derivatives and inspired by mussel-adhesive chemistry represents a promising advance in the field of biomaterials for biomedical applications. This study demonstrates the effective functionalization of micronized graphite via a one-pot, solvent-free 1,3-DCA reaction. The resulting pyrrolidine-functionalized derivatives not only enabled the further incorporation of the catechol group, by reaction with hydrocaffeic acid, but also facilitated the integration of silver nanoparticles, imbuing the material with antibacterial properties.

Hyaluronic acid was modified with the catechol group, through a reaction with dopamine. This modification improved the adhesive properties and crosslinking interactions. A straightforward process of reinforcing the HA-Cat hydrogels with EG derivatives through dispersion in a pH-controlled PBS solution followed by oxidative crosslinking successfully produced composite hydrogels without altering the pore morphology or distribution. SEM analysis confirmed that the porous structure remained suitable for tissue engineering by providing adequate scaffolding for cell infiltration and nutrient diffusion.

Rheological properties demonstrated a frequency-dependent behavior of the hydrogels, with moduli values that align with the requirements for soft-tissue engineering. These results underscore the potential versatility of the hydrogels in mimicking the mechanical environment of native tissues. Additionally, biocompatibility assays, including MTS and live/dead evaluations, confirmed that the composite hydrogels were non-cytotoxic to L929 fibroblast cells, with optimal performance observed at specific concentrations, such as 8.75 mg/mL of HA-Cat hydrogels. The results suggest that these hydrogels hold substantial potential for applications in soft tissue engineering, wound healing, and other biomedical fields requiring multifunctional materials capable of integrating bioadhesive properties, structural reinforcement, and biocompatibility.

## Figures and Tables

**Table 1:** Atomic percentages of the elements present in each sample, as obtained by XPS.

	Atomic %			
	C 1s	O 1s	N 1s	Ag 3d
EG	97.48	2.52	-	-
f-EG	89.43	4.85	5.32	-
f-EG-AgNP	89.45	5.57	4.46	0.52
f-EG-Cat	76.04	15.47	5.25	-

**Table 2:** Different approaches for the reinforcement of HA-Cat hydrogels with f-EG derivatives.

Sample	Method
1	Dissolution of HA-Cat in PBS at pH 8–9, and posterior addition of f-EG to the pre-gel solution
2	Dispersion of f-EG in PBS solution at pH 8–9, followed by the dissolution of the HA-Cat in the dispersion
3	Mixing of the solids of HA-Cat and f-EG, and then the addition of PBS solution at pH 8–9

## Data Availability

Data are contained within the article and Supplementary Materials.

## Supplementary Materials

The following supporting information can be downloaded at: https://www.mdpi.com/article/10.3390/nano15040276/s1. Figure S1: Images of (a) f-EG, (b) F-EG-AgNPs and (c) F-EG-Cat; Figure S2: HA-Cat formulation was prepared by varying the concentration of HA-Cat conjugate and the amount of oxidizing agent, sodium periodate; Table S1: Optimization of different parameters for the preparation of adhesive hyaluronic acid hydrogels; Figure S3: SEM images of the (a) EG and (b) f-EG; Figure S4: High-resolution XPS: (a) EG, (b) f-EG, (c) f-EG-AgNP, and (d) f-EG-Cat; Figure S5: UV-vis spectra of HA-cat (λ = 200–320 nm) with different concentrations. The absorbance is presented in arbitrary units; Figure S6: GPC spectra of the HA and HA-Cat, respectively (See Ref. [39]); Figure S7: Formation of HA-Cat hydrogel after adding NaIO_4_; Figure S8: HA-Cat hydrogels reinforced with f-Micrograf, were prepared through different methods of mixing the filler and the polymeric matrix. Figure S9: Live/dead fluorescence images of L929 fibroblast cells after incubation with cell culture medium that was previously in contact with 35 mg/mL of HA-Cat hydrogels incorporated with different graphene derivatives; Figure S10: L929 fibroblast cells morphology after incubation with cell culture medium that was previously in contact with 35 mg/mL of HA-Cat hydrogels incorporated with different graphene derivatives; Figure S11: Live/dead fluorescence images of L929 fibroblast cells after incubation with cell culture medium that was previously in contact with 17.5 mg/mL of HA-Cat hydrogels incorporated with different graphene derivatives; Figure S12: L929 fibroblast cells morphology after incubation with cell culture medium that was previously in contact with 17.5 mg/mL of HA-Cat hydrogels incorporated with different graphene derivatives; Figure S13: L929 fibroblast cells morphology after incubation with cell culture medium that was previously in contact with 8.75 mg/mL of HA-Cat hydrogels incorporated with different graphene derivatives.
