# Supplementary material for: Mussel-Inspired Hydrogels Incorporating Graphite Derivatives for Soft Tissue Regeneration
Source: Nanomaterials (Basel). 2025 Feb 12;15(4):276. doi: 10.3390/nano15040276 (PMC11858166; doi:10.3390/nano15040276)
Supplement: Supplementary file 1 [file nanomaterials-15-00276-s001.zip › nanomaterials-3430670-supplementary.pdf]

Supporting Information

Mussel-Inspired Hydrogels Incorporating Graphite Derivatives for Soft Tissue Regeneration

Filipa Fernandes [1,2,3,†], Daniela Peixoto [1,2,†], Cátia Correia [1,2], Magda Silva [1,2,3], Maria C. Paiva [3,*], Natália M. Alves [1,2,*]

[1] 3B's Research Group, I3Bs—Research Institute on Biomaterials, Biodegradables and Biomimetics, University of Minho Headquarters of the European Institute of Excellence on Tissue Engineering and Regenerative Medi-cine, Avepark, 4805-694 Guimarães, Portugal; pg38293@alunos.uminho.pt (F.F.); daniela.peixoto@i3bs.uminho.pt (D.P.); catia.correia@i3bs.uminho.pt (C.C.)

[2] ICVS/3B's, Associate PT Government Laboratory, 4710-057 Braga, Portugal

[3] Department of Polymer Engineering, Institute for Polymers and Composites, University of Minho, 4800-058 Guimarães, Portugal; id6877@uminho.pt, pg38293@alunos.uminho.pt

[*] Correspondence: mcpaiva@dep.uminho.pt (M.C.P.); nalves@i3bs.uminho.pt (N.M.A.)

[†] These authors contributed equally to this work.

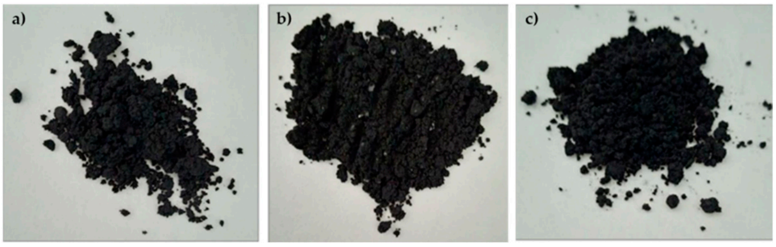

Figure S1. Images of a) f-EG, b) F-EG-AgNPs and c) F-EG-Cat

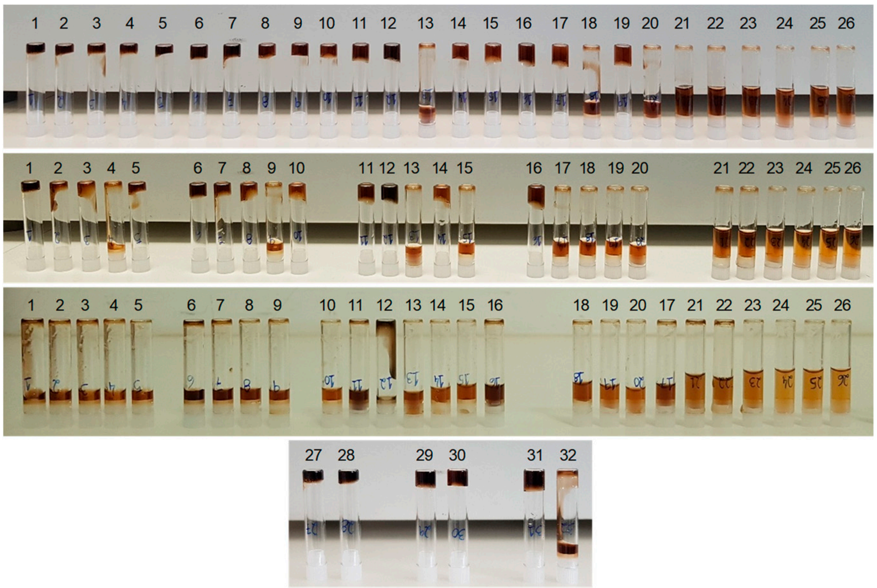

Figure S2. HA-Cat formulation was prepared by varying the concentration of HA-Cat conjugate and the amount of oxidizing agent, sodium periodate.

Table S1. Optimization of different parameters for the preparation of adhesive hyaluronic acid hydrogels.

	HA-Cat (mg)	PBS pH8-9 (μL)	$NaIO_4$ (mg)		HA-Cat (mg)	PBS pH8-9 (μL)	$NaIO_4$ (mg)
sample 1			1	sample 16			1
sample 2			2	sample 17			2
sample 3		100	3	sample 18		250	3
sample 4			4	sample 19			4
sample 5			5	sample 20			5
sample 6			1	sample 21			1
sample 7			2	sample 22			2
sample 8	10	150	3	sample 23		500	3
sample 9			4	sample 24	10		4
sample 10			5	sample 25			5
sample 11			1	sample 26			6
sample 12			2	sample 27		100	0.25
sample 13		200	3	sample 28			0.5
sample 14			4	sample 29		150	0.25
sample 15			5	sample 30			0.5
				sample 31		200	0.25
				sample 32			0.5

Scanning electron microscopy (SEM) images of the EG and f-EG are presented in Figure S2, illustrating the similar morphology before and after functionalization.

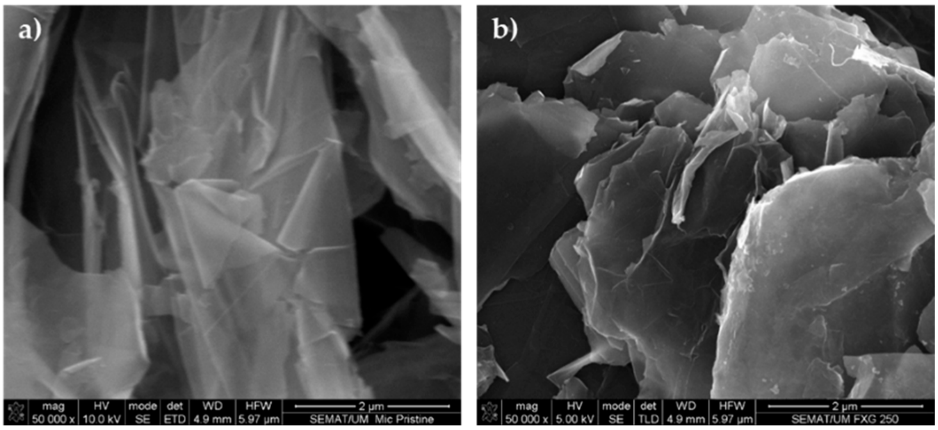

Figure S3. SEM images of the a) EG and b) f-EG.

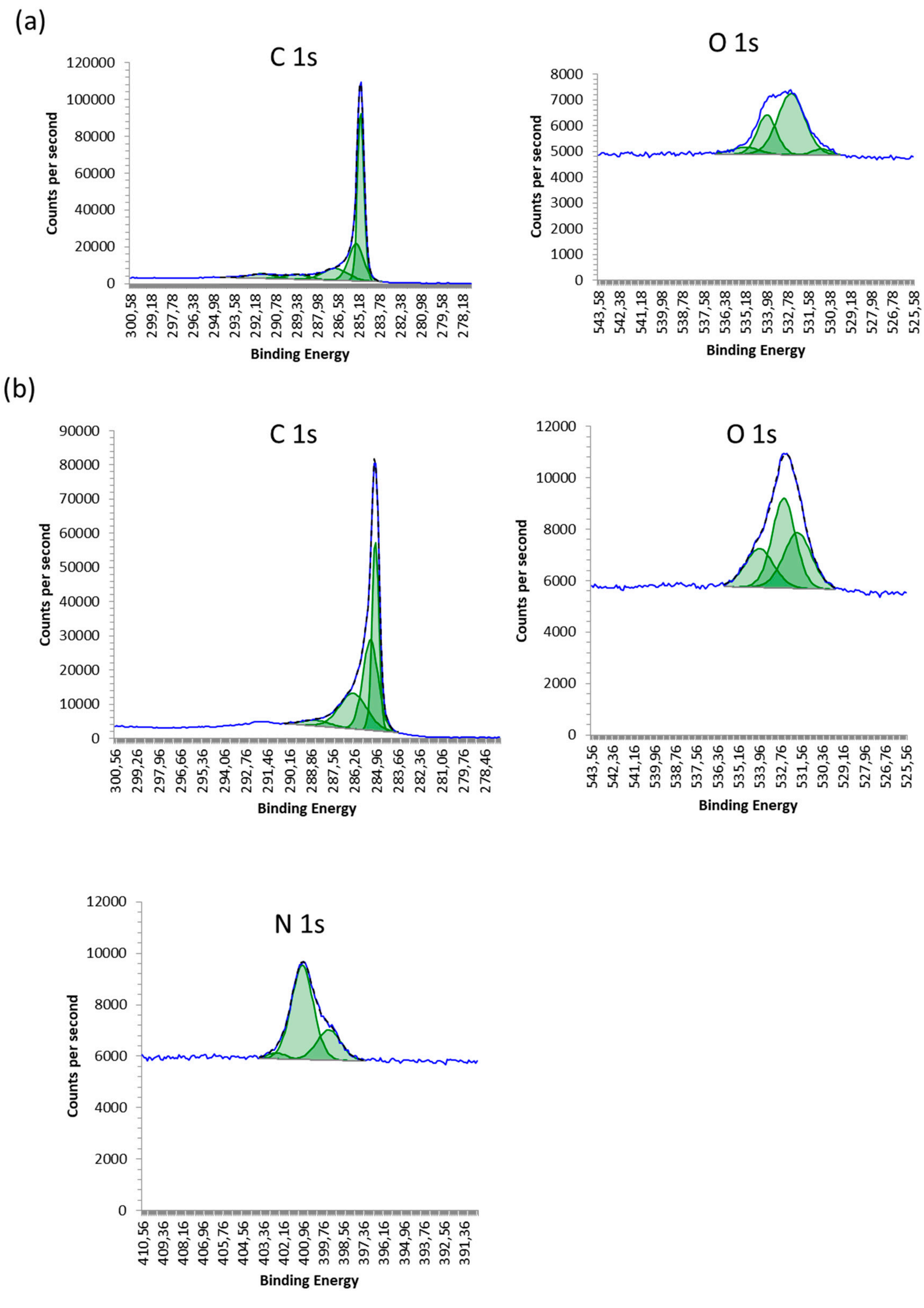
(a)
C 1s
Counts per second
Binding Energy
O 1s
Counts per second
Binding Energy
(b)
C 1s
Counts per second
Binding Energy
O 1s
Counts per second
Binding Energy
N 1s
Counts per second
Binding Energy

(c)

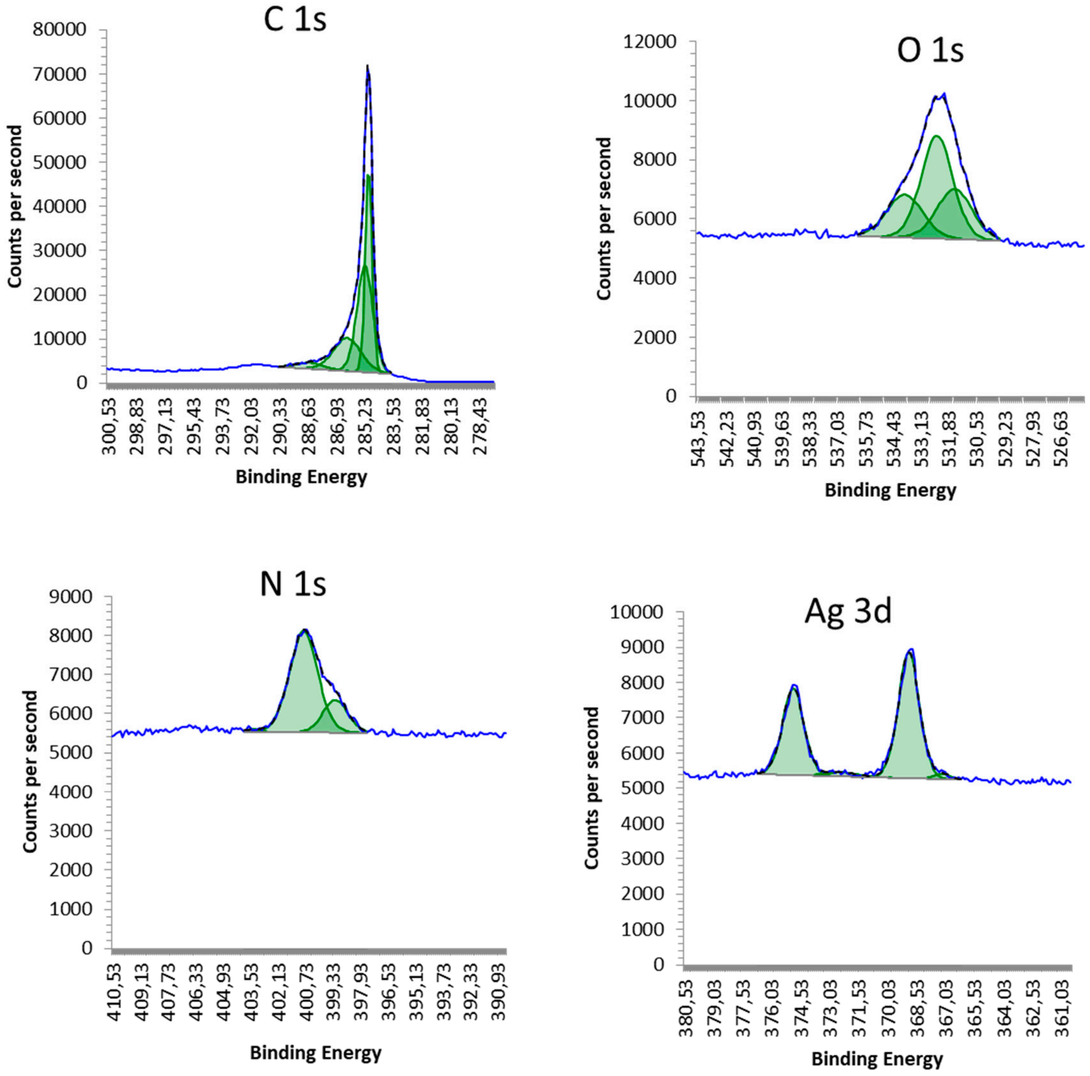

C 1s
Counts per second
Binding Energy
O 1s
Counts per second
Binding Energy
N 1s
Counts per second
Binding Energy
Ag 3d
Counts per second
Binding Energy

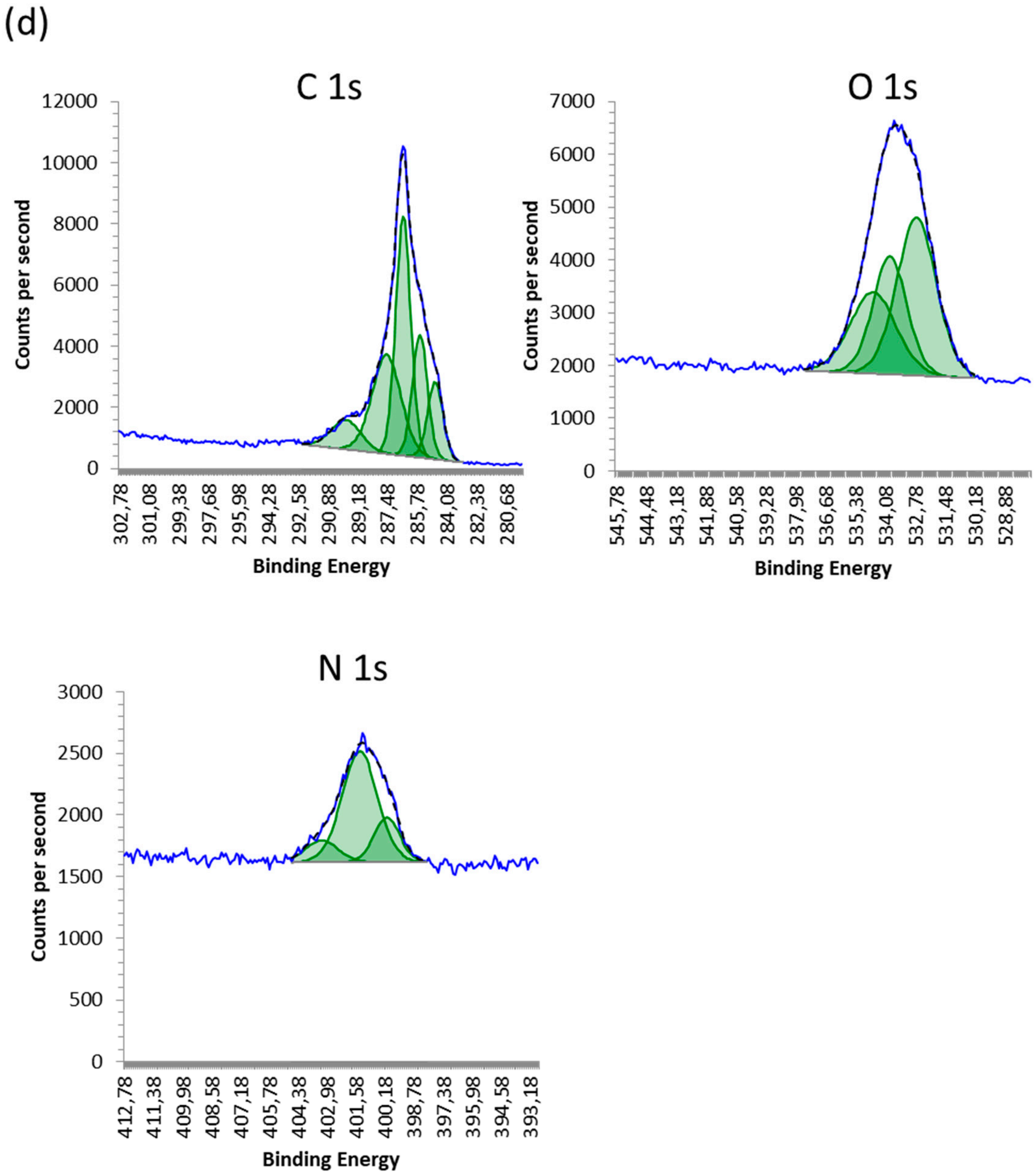

Figure S4. High-resolution XPS: **(a)** EG, **(b)** f-EG, **(c)** f-EG-AgNP, and (**d**) f-EG-Cat.

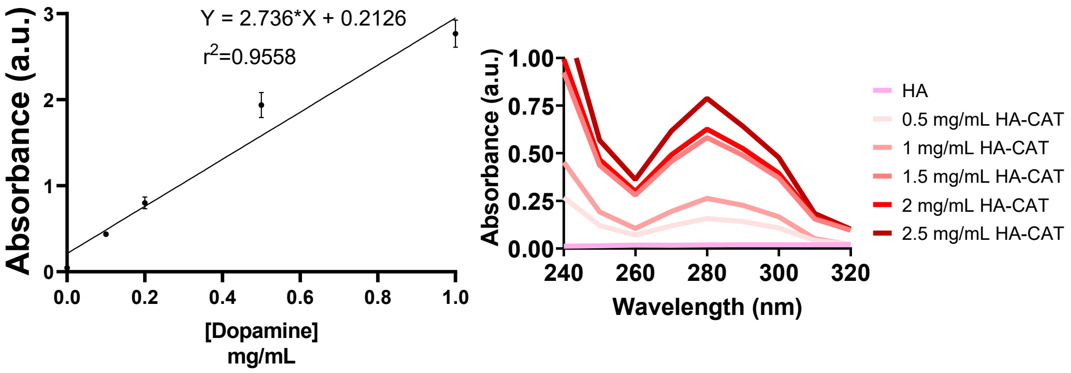

Figure S5. UV-vis spectra of HA-cat (λ=200-320 nm) with different concentrations. The absorbance is presented in arbitrary units.

Gel permeation Chromatography (GPC)

GPC measurements were performed with a Malvern Viscotek TDA 305 with refractometer (RI-Detector 8110, Bischoff), right and low angle light scattering (LS) and viscometer detectors on a set of four columns: pre-column Suprema, 5 µm, 8x50, Suprema 30 Å, 5 µm, 8x300, and 2x Suprema 1000 Å, 5 µm 8x300. The system was kept at 30 °C. We have used PBS buffered Saline (0.01 M phosphate buffer, 0.0027 M potassium chloride and 0.137 M sodium chloride, pH 7.4, at 25 °C, Sigma-Aldrich) and 0.05% w/v NaN_3 at rate of 1 mL min^{-1}. The absolute molecular weight was determined by a calibration of the RI and LS detectors performed using the software Omnisec 5.12 (ViskoteK) with a pullulan of Mn 48.8 kDa and PDI 1.07. The dn/dc of HA was taken from the literature [39].

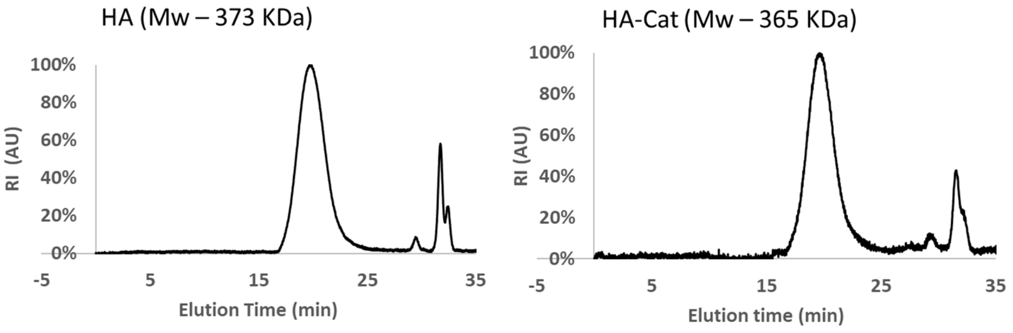

Figure S6. GPC spectra of the HA and HA-Cat, respectively

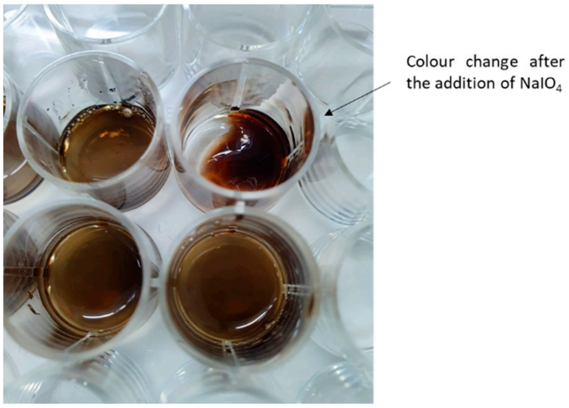

Figure S7. Formation of HA-Cat hydrogel after adding $NaIO_4$

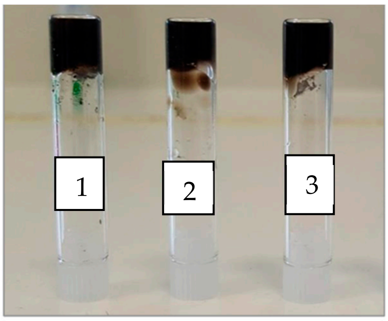

Figure S8. HA-Cat hydrogels reinforced with f-Micrograf, were prepared through different methods of mixing the filler and the polymeric matrix.

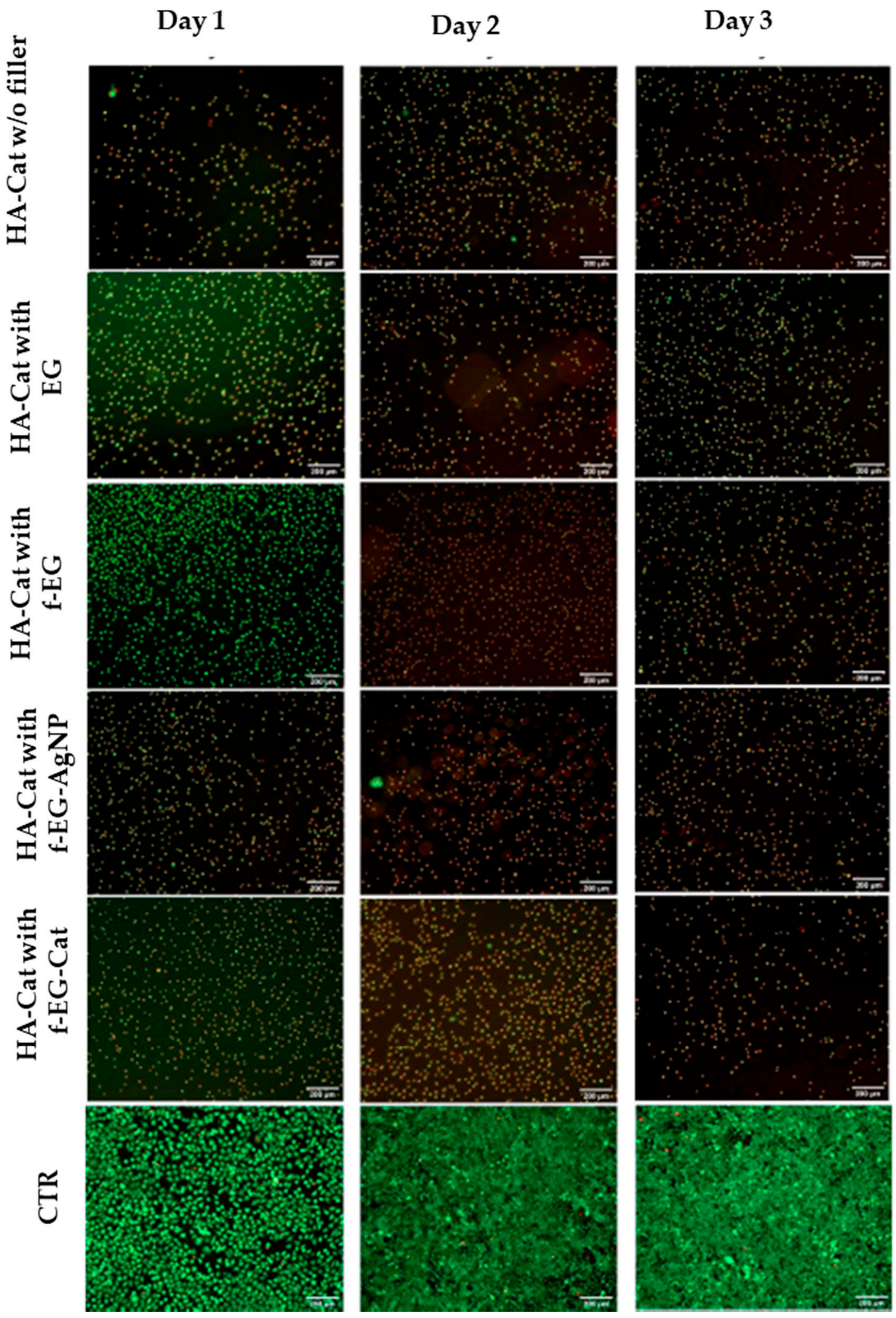

Figure S9. Live/dead fluorescence images of L929 fibroblast cells after incubation with cell culture medium that was previously in contact with 35mg/ml of HA-Cat hydrogels incorporated with different graphene derivatives.

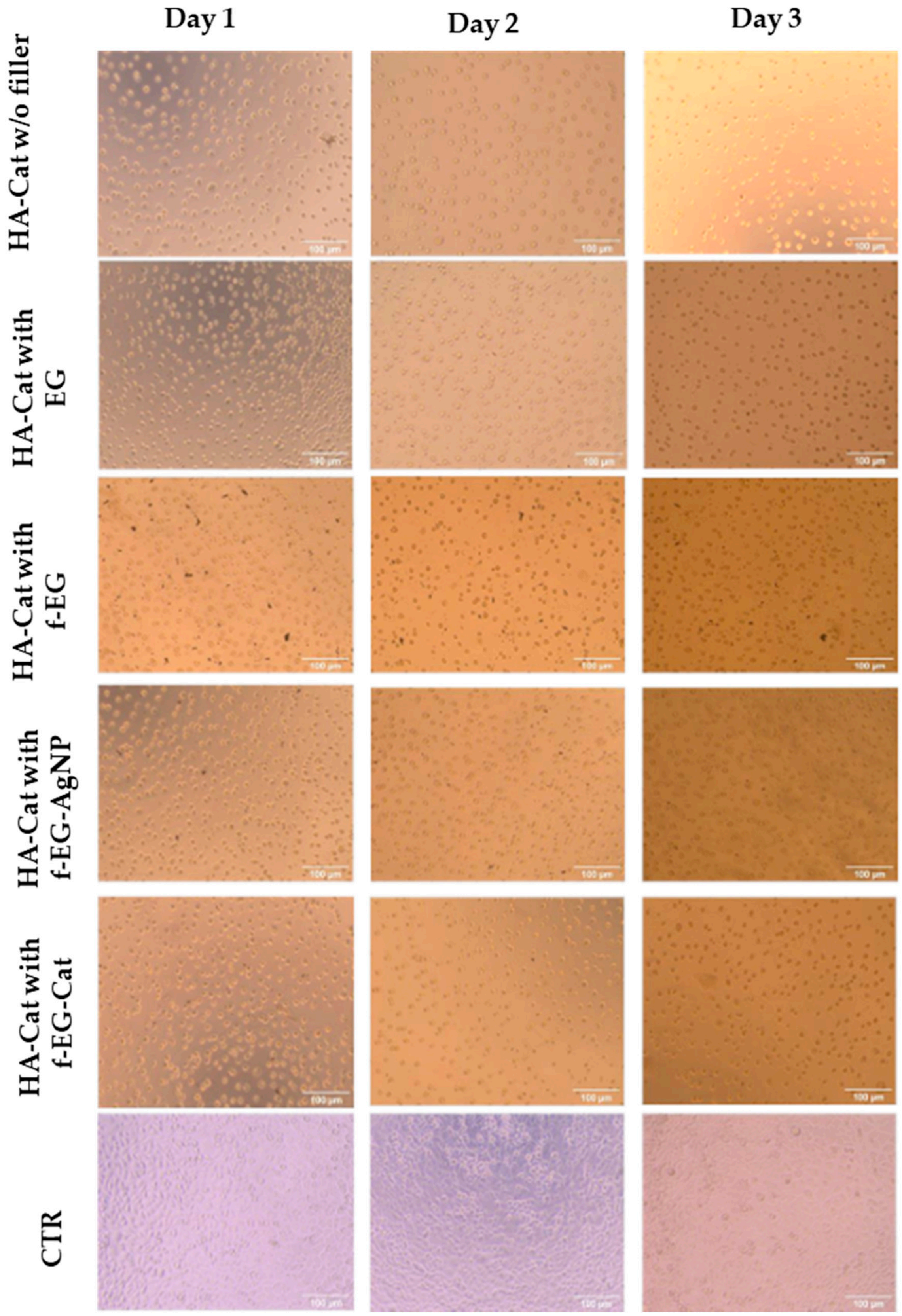

Figure S10. L929 fibroblast cells morphology after incubation with cell culture medium that was previously in contact with 35mg/ml of HA-Cat hydrogels incorporated with different graphene derivatives.

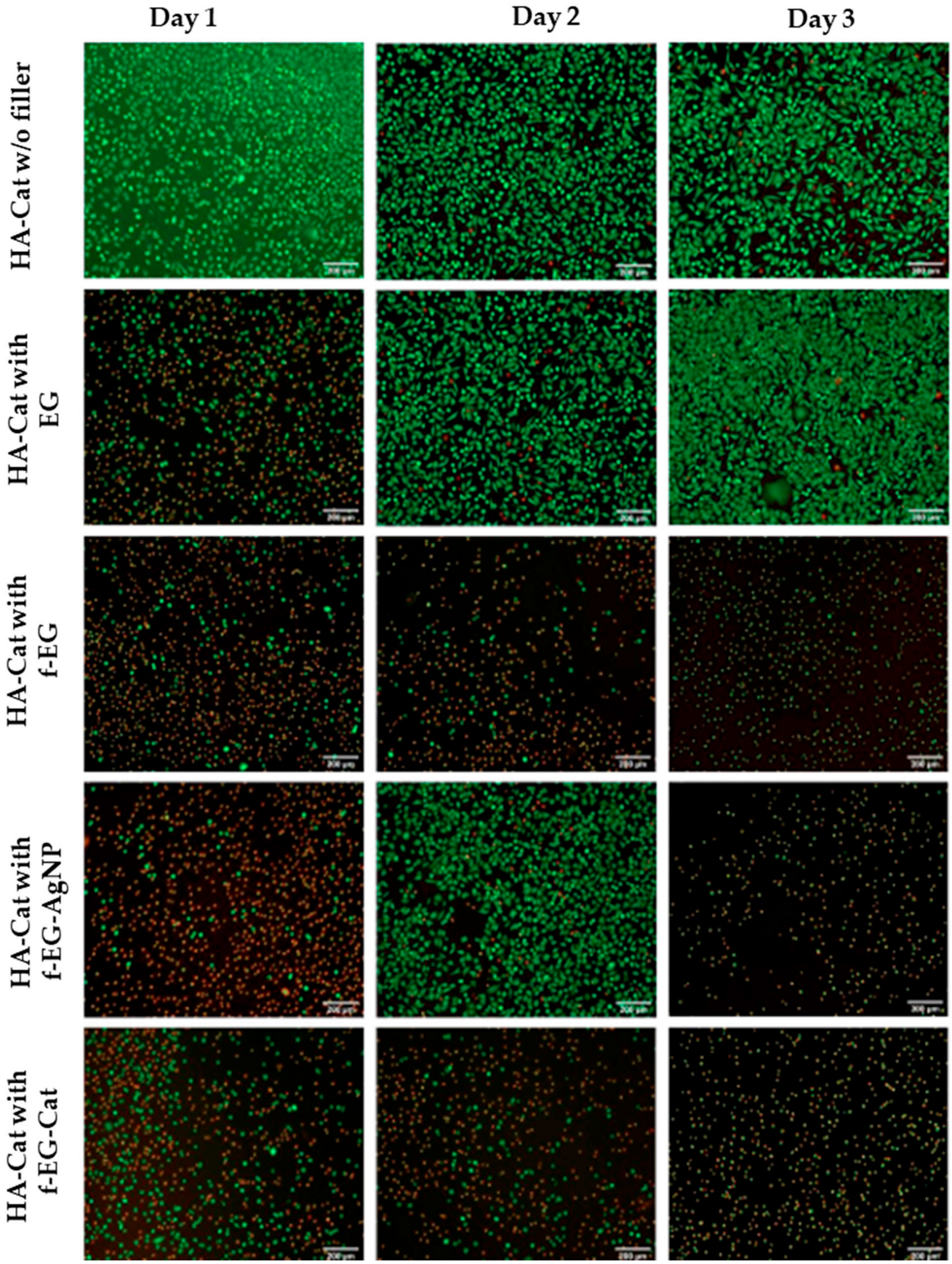

Figure S11. Live/dead fluorescence images of L929 fibroblast cells after incubation with cell culture medium that was previously in contact with 17.5mg/ml of HA-Cat hydrogels incorporated with different graphene derivatives.

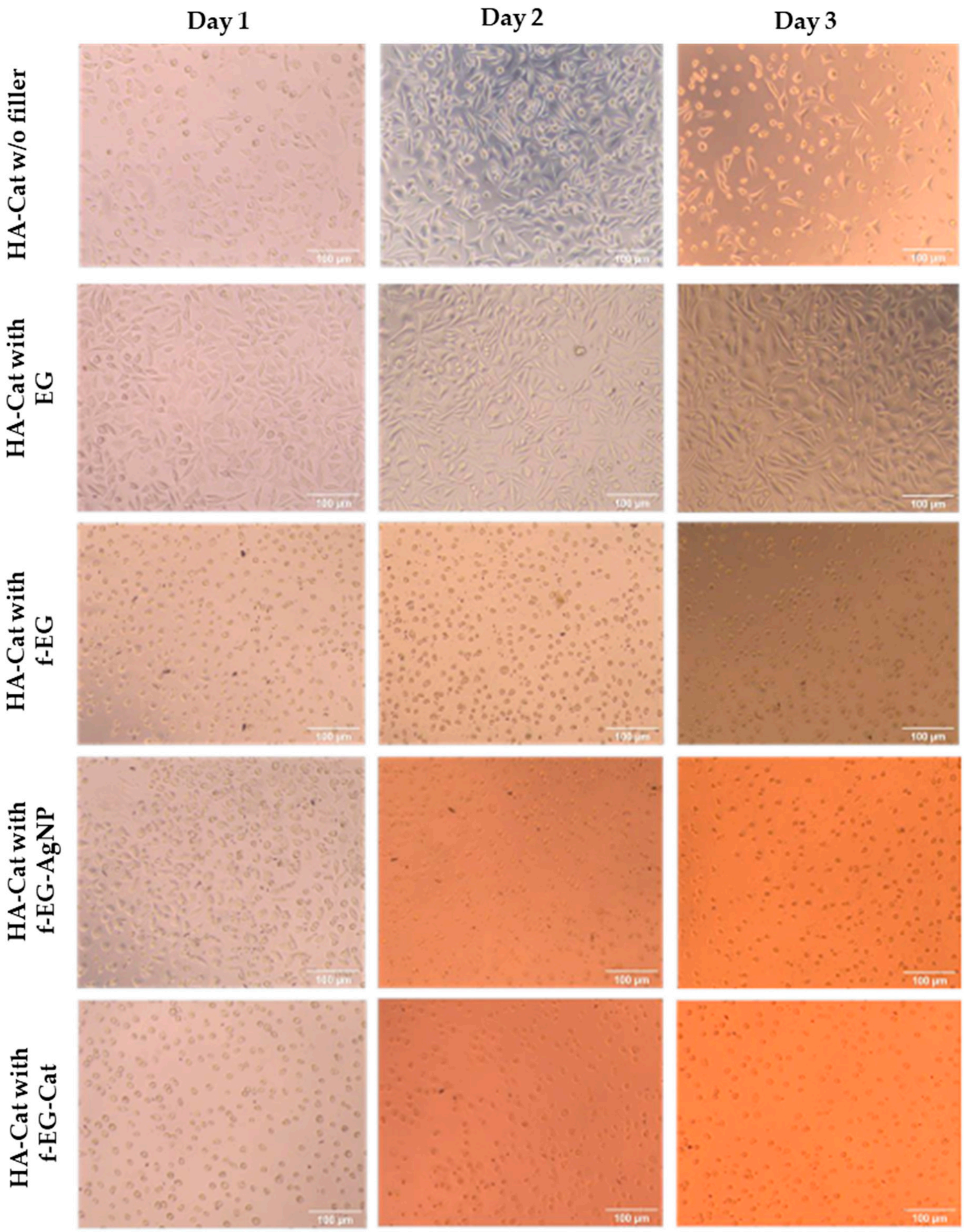

Figure S12. L929 fibroblast cells morphology after incubation with cell culture medium that was previously in contact with 17.5mg/ml of HA-Cat hydrogels incorporated with different graphene derivatives.

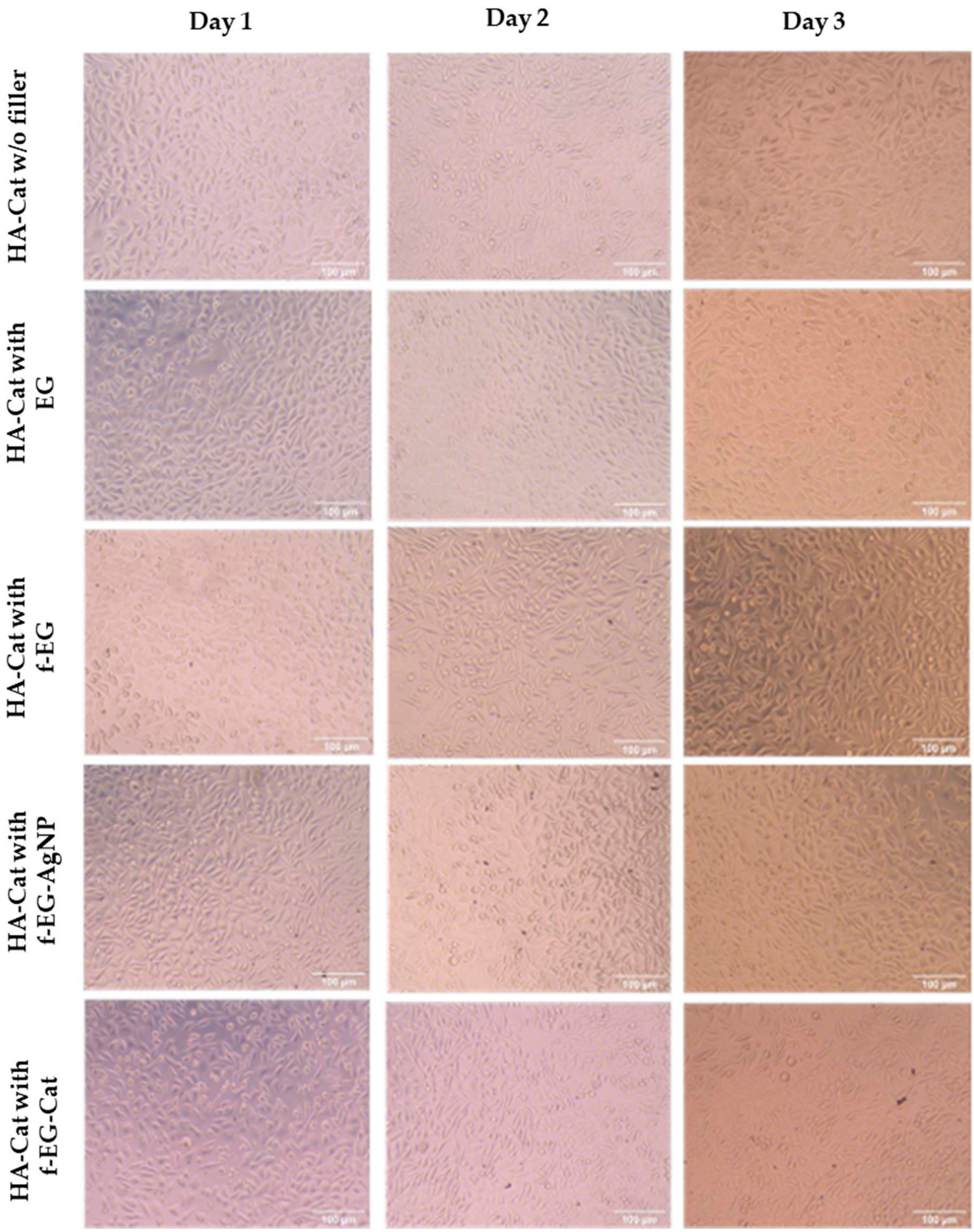

Figure S13. L929 fibroblast cells morphology after incubation with cell culture medium that was previously in contact with 8.75mg/ml of HA-Cat hydrogels incorporated with different graphene derivatives.
